# Identifying Key Factors for Burnout Among Orthopedic Surgeons Using the Analytic Hierarchy Process Method

**DOI:** 10.3389/ijph.2023.1605719

**Published:** 2023-05-03

**Authors:** Shiqian Wang, Lin Li, Yanjun Jin, Rui Liao, Yen-Ching Chuang, Zhong Zhu

**Affiliations:** ^1^ Business School, Dongguan City University, Dongguan, China; ^2^ Taizhou Hospital of Zhejiang Province Affiliated to Wenzhou Medical University, Taizhou, China; ^3^ Business College, Taizhou University, Taizhou, Zhejiang, China; ^4^ Institute of Public Health and Emergency Management, Taizhou University, Taizhou, Zhejiang, China; ^5^ Key Laboratory of Evidence-Based Radiology of Taizhou, Linhai, Zhejiang, China; ^6^ Department of Orthopaedics, Taizhou Hospital of Zhejiang Province Affiliated to Wenzhou Medical University, Taizhou, China

**Keywords:** burnout, multiple criteria decision-making (MCDM), orthopedic surgeons, analytic hierarchy process (AHP), key factors

## Abstract

**Objectives:** To develop an evaluation model for, and identify key factors contributing to, burnout in orthopedic surgeons, providing a reference for the management of burnout among orthopedic surgeons in hospitals.

**Methods:** We developed an analytic hierarchy process (AHP) model with 3 dimensions and 10 sub-criteria based on an extensive literature review and expert assessment. We used expert and purposive sampling and 17 orthopedic surgeons were selected as research subjects. The AHP process was then used to obtain the weights and to prioritize the dimensions and criteria for burnout in orthopedic surgeons.

**Results:** The dimension of *C*_1_ (personal/family) was the key factor affecting burnout in orthopedic surgeons, and in the sub-criteria, the top four sub-criteria were *C*_11_ (little time for family), *C*_31_ (anxiety about clinical competence), *C*_12_ (work-family conflict), and *C*_22_ (heavy work load).

**Conclusion:** This model was effective in analyzing the key factors contributing to job burnout risk, and the results can inform improved management of the levels of burnout affecting orthopedic surgeons in hospitals.

## Introduction

Burnout is an important concept in occupational stress and mental health research and was first proposed by Freudenberg in 1974 (1). Since then, research on job burnout has received increasing attention, and burnout in different occupations has become a research hotspot in the field of occupational stress, including physician burnout (2), faculty burnout (3), and nurse burnout (4). Job burnout is defined as a comprehensive reaction marked by extreme physical and mental exhaustion caused by occupational stress, and it is characterized by two features: one key aspect is the negative, cynical attitude and feelings toward clients, and the other is an increase in emotional exhaustion (5).

Physician burnout has become one of the core topics in occupational stress research in the medical field (6–8). This research has identified physician burnout as a public health problem (9), especially in the context of the current COVID-19 pandemic (10, 11). Physicians and nurses typically help others and are generally under heavy work pressure, and some of them even suffer from depression as a result (12, 13). Therefore, discussing job burnout among physicians is of great practical significance.

Studies have shown that during the current pandemic, the occupational stress of physicians has increased significantly (14). Stress is a state of increased tension as a protective reaction to various adverse factors. At the same time, emotions are a mental process that reflects the attitude towards these factors. Burnout caused by negative emotions, such as anxiety, anger, depression and sadness, affects the physical and mental health of physicians (15, 16) and can decrease their career (9, 16) and life satisfaction (13, 17) among other factors. This can have significant adverse effects on the medical system; for example, burnout has been shown to lead to medical malpractice (18, 19) and to reduce the quality of medical treatment patients receive (20, 21).

Orthopedic surgery is a challenging profession that often involves a heavy workload, generally long working hours, and requires a wide range of medical knowledge and specialist surgical skills (22, 23). Research has shown that half of orthopedic surgeons report experiencing burnout (24, 25). Overall, orthopedic surgeons had the second highest burnout rate (trauma surgeons ranked first) (26). However, there were discrepancies between burnout level for orthopedic surgeons and physicians in other medical specialties (15). Based on this review of the relevant literature, this study aimed to identify the main factors affecting burnout in orthopedic surgeons and to build a hierarchy model. To achieve this, we designed and conducted a questionnaire survey and elicited responses from 17 orthopedic surgeons. The questionnaire results were summarized and discussed using the analytic hierarchy process (AHP) to obtain the weight order of each criterion in pairwise comparisons (27). Subsequent to this, hospital decision-makers can further focus on the primary factors contributing to orthopedic physicians’ burnout, and implement corresponding policy measures to improve their working conditions and enhance the hospital’s management level.

## Methods

### Study Design and Modeling Process

**FIGURE 1:**
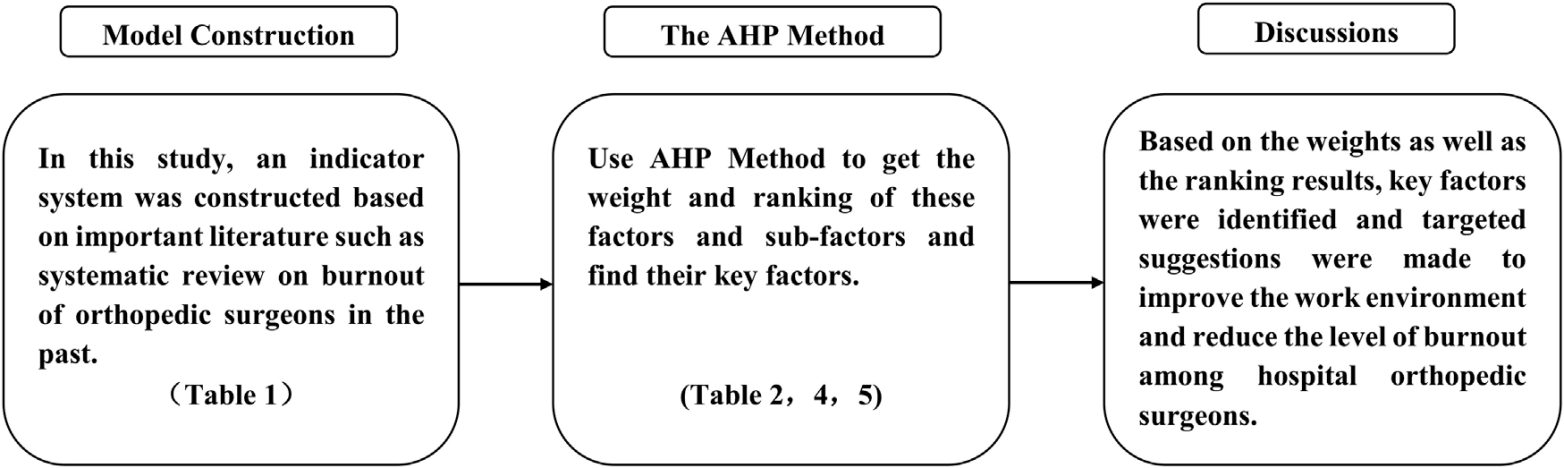
Study flow chart (China, 2023).

This study constructs an indicator system by reviewing past systematic reviews and other important literature on occupational burnout among orthopedic surgeons. We then obtain the weights and rankings of these factors and sub-factors through a survey of 17 orthopedic physicians using the AHP method, identifying the critical factors. Please see Figure 1 for the flowchart.

### The Model of Burnout Factors for Orthopedic Surgeons

This study builds on multiple previous studies to identify key factors that influence orthopedic surgeon burnout. Existing research has used a variety of methods to explore this topic such as systematic reviews, content analyses, meta-analyses, and case studies. For example, the Maslach Burnout Inventory is a widely used measure and is considered the gold standard for burnout assessment. It is a scoring system validated across multiple occupations and fields of study and includes three components: burnout, depersonalization, and personal achievement (28).

Sibeoni and Bellon-Champel (29) analyzed the factors related to physician burnout through a systematic review and meta-synthesis method, and divided these factors into two themes: the stress factor and protective factor. The stress factor theme initially considered organizational factors, then relationship factors, and finally personal factors. Travers (30) concluded in a review article that there were six relevant factors affecting burnout in orthopedic surgeons but highlighted the two most significant factors as the intensity and complexity of the work and its significant emotional requirements. Hui and Leung (31) analyzed the positive and negative factors of occupational burnout among orthopedic surgeons using a systematic review method that focused on four factors: personal, family, work environment, and occupation. Verret et al. (32) used a simple linear regression model to summarize burnout factors in orthopedic surgeons at different career stages. Through an open-comment analysis, they identified five major problematic categories in work life: workload, resources, interaction, environment, and self-care. Based on the above key articles, this study constructed a model of burnout factors for orthopedic surgeons, and its related factors and sub-factors are shown in Table 1.

**TABLE 1:** The burnout factors for orthopedic surgeons (China, 2023).

Criteria	Sub-criteria	References
C_1_ Personal/family factors	C_11_ Little time for family	(21)
	C_12_ Work-family conflict	(33, 34)
	C_13_ Lack of spousal support/poor marital relationship	(33, 35)
C_2_ Working environment	C_21_ Sleep deprivation	(35)
	C_22_ Heavy work load	(34, 35)
	C_23_ Perception of stress in work	(34)
	C_24_ Stress in workplace relationships	(34)
C_3_ Career	C_31_ Anxiety about clinical competence	(34)
	C_32_ Worry about competition from other orthopedic surgeons	(34)
	C_33_ Precariat of some doctors’ status, associated with substantial concern about their future	(36–38)

### The Analytic Hierarchy Process Method

The AHP is a subjective evaluation method proposed by Saaty, an American operational research scientist, in the early 1970s (39). This is a multi-attribute evaluation method used under certain conditions. The AHP is a systematic, simple, flexible, and effective decision-making method that systematizes complex problems using a hierarchical structure. This method decomposes the elements related to decision-making into multiple levels, such as objectives, criteria, and schemes and conducts qualitative and quantitative analysis on this basis (27). The AHP has the advantage of simplifying complex problems and calculations. It is widely used in many fields such as personnel quality evaluation (40), multi-scheme comparison (41), scientific and technological achievement evaluation (42), and work effectiveness evaluation (43). The AHP mainly includes four steps (27): the first step is the construction of the hierarchical structure model; the second step is the construction of the judgment matrix; the third step is testing the hierarchical single ordering and its consistency (this determines the weight of indicators); and the fourth step is the hierarchical total ordering and its consistency test.

Step 1 Obtain the paired comparison matrix from each expert.

Each expert applied a 9-point Likert scale (from equally important to absolutely important) to assess the degree of relative importance between criteria, as shown in Eq. 1.

R=r11…r1j…r1n⋮⋱⋮⋱⋮ri1…rij…rin⋮⋱⋮⋱⋮rn1…rnj…rnnn×n=1…r1j…r1n⋮⋱⋮⋱⋮1r1j…1…rin⋮⋱⋮⋱⋮1r1n…1rin…1n×n (1)

where n is the number of criteria; and rji=1rij (positive reciprocal).

Step 2 Calculate the relative weight between criteria.

The approximate weight of each criterion is obtained by the maximum eigenvalue of the eigenvector, which is given by Eq. 2.

$$w_{i}=\frac{{\left(\prod_{j=1}^{n}r_{ij}\right)}^{\frac{1}{n}}}{\sum_{i=1}^{n}{\left(\prod_{j=1}^{n}r_{ij}\right)}^{\frac{1}{n}}}i,j=1,2,\ldots ,n$$ (2)

Step 3 obtain the consistency of pairwise matrix.

Because the decision maker’s judgment matrix is irrational, we can use Eqs 3, 4 to calculate the deviation degree of judgment inconsistency, which is called the consistency index (CI) and consistency ratio (CR).

$$CI=\frac{\lambda_{\max}-n}{n-1}$$ (3)

$$CR=\frac{CI}{RI}$$ (4)

where $\lambda_{\max}$ is the maximum eigenvalue of the matrix R ; the random consistency (RI) index is shown in Table 2.

If the C.R. ≤ 0, the judgment is completely consistent. If the C.R. is between 0 and 0.1, it means that the judgment is not completely consistent, but it is still within the acceptable deviation range. If the C.R. ≤ 0.1, the judgment is inconsistent.

**TABLE 2:** Random index (RI) (United States, 1987).

Order	1	2	3	4	5	6	7	8	9	10	11	12	13	14	15
RI	—	—	0.58	0.90	1.12	1.24	1.32	1.41	1.45	1.49	1.51	1.54	1.56	1.57	1.58

### Data Collection

The study was conducted between 18 March and 20 April 2022, and each questionnaire took 20–30 min. The participants in the study were all orthopedic surgeons with higher education, extensive clinical experience, and good professional knowledge. With 17 participants, all from the Department of Orthopedics of Taizhou Hospital of Zhejiang Province, this study met the requirements of the AHP analysis method.

All participants were male; 13 participants were between the ages of 30 and 39, accounting for 76.4%; 2 participants were under the age of 30 and 2 over the age of 40, each category accounting for 11.8%. The participants were highly educated: all of them had a bachelor’s degree, while 64.7% had a master’s degree or above. Only four participants had less than 10 years of work experience, and 76.5% had more than 10 years of work experience. From the job title perspective, 64.7% of the participants had director physician titles and, of the remaining 35.3%, two participants were assistant director physicians and two were physicians. Specific descriptive information is shown in Table 3.

**TABLE 3:** Background and description of 17 orthopedic clinicians (China, 2023).

Characteristics	Value (%)
Gender	
Male	17 (100%)
Female	0 (0%)
Age	
<30	2 (11.8%)
30–39	13 (76.4%)
40–49	2 (11.8%)
Education	
Bachelor	6 (35.3%)
Master or above	11 (64.7%)
Years of service	
Under 10 years	4 (23.5%)
10–15	10 (58.8%)
15–20	3 (17.7%)
Professional title	
Director Physician	11 (64.6%)
Assistant Director Physician	2 (11.8%)
Physician	2 (11.8%)
Other	2 (11.8%)

## Results

In our study, we took the first orthopedic surgeon as an example for calculation and filled in the original paired matrix according to the AHP method. The CI and CR values of the original paired matrix of the criteria and sub-criteria were all less than 0.1, their consistency passed the test, and the results are shown in Table 4.

**TABLE 4:** Paired comparison matrix and local weight results for the first orthopedic surgeon (China, 2023).

Dimensions	*C*_1_	*C*_2_	*C*_3_		Local weight	
C_1_	1	3	6		0.655	C.I. = 0.009
C_2_	1/3	1	3		0.250	C.R = 0.016
C_3_	1/6	1/3	1		0.095	
*C*_1_	*C*_11_	*C*_12_	*C*_13_			
C_11_	1	3	6		0.635	C.I. = 0.047
C_12_	1/3	1	5		0.287	C.R = 0.081
C_13_	1/6	1/5	1		0.078	
*C*_2_	*C*_21_	*C*_22_	*C*_23_	*C*_24_		
C_21_	1	1/5	1/4	1/3	0.072	C.I. = 0.068
C_22_	5	1	3	2	0.471	C.R = 0.076
C_23_	4	1/3	1	1/3	0.164	
*C*_24_	3	1/2	3	1	0.293	
*C*_3_	*C*_31_	*C*_32_	*C*_33_			
C_31_	1	5	4		0.674	C.I. = 0.043
C_32_	1/5	1	1/3		0.101	C.R = 0.074
C_33_	1/4	3	1		0.226	

Table 5 shows the weight of the results of 17 orthopedic surgeons, and the CR and CI values are both less than 0.1, indicating that the constructed matrix has passed the consistency test, and the results include weight ordering of criteria and sub-criteria, and the results are analyzed as follows: From the perspective of local weights, the criteria weight is ranked *C*_1_ (personal/family factors) ≻ *C*_3_ (Career) ≻ *C*_2_ (working environment) from high to low. In the *C*_1_ (personal/family factors) sub-criteria, the weight is ranked from C_11_ (little time for family) ≻ *C*_12_ (work-family conflict) ≻ *C*_13_ (lack of Spousal support/poor marital relationship); In the *C*_2_ (working environment) sub-criteria, the weight is ranked from *C*_22_ (heavy work load) ≻ *C*_23_ (perception of stress in work) ≻ *C*_24_ (stress in workplace relationships) ≻ *C*_21_ (sleep) deprivation); In the C3 (career) sub-criteria, their weight from high to low is ranked from *C*_31_ (anxiety about clinical competence) ≻ *C*_33_ (career development concerns) ≻ *C*_32_ (colleagues competition).

**TABLE 5:** The local weight and global weight results for 17 orthopedic surgeons (China, 2023).

	Dimensions			Criteria									
No.	*C*_1_	*C*_2_	*C*_3_	*C*_11_	*C*_12_	*C*_13_	*C*_21_	*C*_22_	*C*_23_	*C*_24_	*C*_31_	*C*_32_	*C*_33_
1	0.655	0.250	0.095	0.635	0.287	0.078	0.072	0.471	0.164	0.293	0.674	0.101	0.226
2	0.637	0.258	0.105	0.614	0.268	0.117	0.071	0.514	0.179	0.236	0.717	0.088	0.195
3	0.669	0.243	0.088	0.637	0.258	0.105	0.071	0.514	0.179	0.236	0.627	0.280	0.094
4	0.682	0.236	0.082	0.637	0.258	0.105	0.076	0.513	0.150	0.261	0.696	0.075	0.229
5	0.559	0.089	0.352	0.594	0.249	0.157	0.161	0.437	0.093	0.309	0.280	0.094	0.627
6	0.101	0.226	0.674	0.594	0.249	0.157	0.073	0.554	0.126	0.248	0.101	0.226	0.674
7	0.627	0.094	0.280	0.528	0.333	0.140	0.526	0.242	0.109	0.123	0.287	0.078	0.635
8	0.594	0.157	0.249	0.594	0.249	0.157	0.487	0.230	0.104	0.180	0.280	0.094	0.627
9	0.655	0.250	0.095	0.594	0.249	0.157	0.068	0.567	0.123	0.242	0.648	0.230	0.122
10	0.258	0.105	0.637	0.594	0.249	0.157	0.051	0.565	0.134	0.250	0.627	0.094	0.280
11	0.637	0.258	0.105	0.594	0.157	0.249	0.496	0.267	0.083	0.154	0.709	0.179	0.113
12	0.105	0.258	0.637	0.105	0.637	0.258	0.076	0.513	0.261	0.150	0.287	0.078	0.635
13	0.297	0.540	0.163	0.709	0.179	0.113	0.092	0.250	0.480	0.177	0.333	0.333	0.333
14	0.297	0.540	0.163	0.594	0.249	0.157	0.093	0.309	0.437	0.161	0.333	0.333	0.333
15	0.333	0.333	0.333	0.669	0.243	0.088	0.094	0.371	0.371	0.163	0.594	0.249	0.157
16	0.117	0.268	0.614	0.101	0.226	0.674	0.044	0.236	0.603	0.116	0.455	0.091	0.455
17	0.717	0.088	0.195	0.143	0.429	0.429	0.094	0.371	0.371	0.163	0.637	0.258	0.105
Average (Local weight)	0.467	0.247	0.286	0.525	0.281	0.194	0.156	0.407	0.233	0.204	0.487	0.169	0.343
Ranking	1	3	2	1	2	3	4	1	2	3	1	3	2
Global weight	0.245	0.131		0.091	0.038	0.100	0.058		0.050	0.140	0.049		0.098
Ranking	1	3		6	10	4	7		8	2	9		5

The global weight of each sub-criterion is calculated by multiplying its local weight with the corresponding criteria’s local weight along the AHP hierarchy. In terms of the global weight of each sub-criteria, the top four sub-criteria are *C*_11_ (little time for family), *C*_31_ (anxiety about clinical competence), *C*_12_ (work-family conflict) and *C*_22_ (heavy work load).

## Discussion

### Clinical Research Implications

Based on the weight results in the previous section, from the perspective of local weight, the weight value of personal and family factors is 0.467, which is close to 0.5, indicating that personal and family factors are the key factors causing burnout of orthopedic surgeons and have a high degree of influence (44). From the perspective of global weight, the top four sub-criteria are *C*_11_, *C*_31_, *C*_12_, and *C*_22_, these factors are little time for family, anxiety about clinical competence, work-family conflict, and heavy work load.

Among the criteria of personal and family, the sub-criteria influencing burnout of orthopedic surgeons were little time for family and work-family conflict, with weights of 0.245 and 0.140. Orthopedic surgeons believe that the workday is too long, there is no time for their personal lives, and they struggle to balance their personal and work lives (45–47). According to Agana research, working hours are the most important factor affecting orthopedic surgeons’ satisfaction, accounting for 62% of the overall job satisfaction (48). Studies suggest that work-family conflict can seriously affect the burnout level of orthopedic surgeons (35), and that frequent conflict leads to poor marital relationships, which further increases the burnout level (34).

The local weight of career and working environment is 0.247 and 0.286. The global weight of the sub-criteria of anxiety about clinical competence in the career criteria, ranks the second. Meanwhile, it can be seen that the local weight value of anxiety about clinical competence is also high, which is 0.487, the highest value of all local weights. It can also be concluded that anxiety about clinical competence has a significant impact on orthopedic surgeon burnout (46).

In the dimension of the working environment, the key sub-criterion is work overload, which has a global weight of 0.1. Work overload is a major source of exhaustion and, in turn, is at the root of burnout. Work overload represents the basic individual stress component of burnout (49), and has high impact on burnout among orthopedic surgeons (50). Continuously increasing responsibilities and extremely high workload were commonly reported problems (32, 36), especially at this stage of the COVID-19 pandemic, the additional workload has led to higher levels of burnout among orthopedic surgeons (51). Workloads also include administrative workloads, such as excessive paperwork, electronic medical records (52), and conference report (53), which also affects their burnout levels.

### Clinical Practice Implications

The general burnout rate among physicians is approximately 40% (44, 54) and the burnout rate of orthopedic surgeons is nearly 55% higher than this general level (55). Furthermore, a study has shown that the burnout rate among Chinese orthopedic surgeons is as high as 85% (24). High burnout rates indicate that these orthopedic physicians face various stressors, which may even cause them to lose their temper at work or develop various health risks, such as heart disease and stroke (24, 56). These issues can severely affect the quality of medical care and potentially lead to an increase in medical errors (19, 57).

According to this study, personal and family factors were key factors in contributing to burnout among orthopedic surgeons. This indicates that, in line with previous findings, reducing working hours to allow orthopedic surgeons to spend time with their families would be an effective way to reduce burnout levels (57, 58), increase happiness at work, and avoid family conflict (48).

Among the factors in the working environment, workload is positively correlated with working time. Therefore, in addition to reducing working hours, it is necessary to improve the efficiency of orthopedic surgeons, especially by reducing administrative workload, reducing the number and length of mandatory meetings, and avoiding bureaucracy (53). To achieve this, system and process transformation are needed, such as upgrading work structure and processes with new technology and simplifying the internal electronic resume platform in hospitals (17).

### Limitations

Our study presents several limitations. First, the investigation builds upon previous research to establish an assessment model for factors contributing to orthopedic physicians’ burnout. These prior studies employed diverse methodologies and, to some extent, were subjective. Second, while our model incorporated key factors identified in previous research, not all factors were compared pairwise due to methodological constraints. Third, past studies were based on correlation, regression, and structural equation models, whereas our results stem from an AHP method, implying that comparisons with many earlier findings may be challenging. Fourth, the participants comprises orthopedic physicians from a specific hospital, suggesting that the results may not be generalizable to other medical professionals in different hospitals. Lastly, due to limitations in the research methodology and participants, factors such as model construction, gender differences among participants, and others were not fully addressed, providing potential avenues for future research.

### Conclusion

In this study, 17 orthopedic surgeons were selected as research subjects to construct an evaluation model of occupational burnout factors. The AHP method was used to obtain weighted results for the factors affecting burnout in orthopedic surgeons, key factors were identified, and improvement strategies were discussed. The results showed that the personal family factor was the key factor affecting burnout in orthopedic surgeons, and in the sub-criteria, the key factors were little time for family, anxiety about clinical competence, work-family conflict, and heavy workload. This research model enriches our understanding of burnout among orthopedic surgeons, and the research results have a certain reference value for informing efforts to reduce burnout rates among orthopedic surgeons.
